# *Fasciola* spp. in Southeast Asia: A systematic review

**DOI:** 10.1371/journal.pntd.0011904

**Published:** 2024-01-17

**Authors:** Vinh Hoang Quang, Bruno Levecke, Dung Do Trung, Brecht Devleesschauwer, Binh Vu Thi Lam, Kathy Goossens, Katja Polman, Steven Callens, Pierre Dorny, Veronique Dermauw

**Affiliations:** 1 Department of Parasitology, National Institute of Malariology, Parasitology and Entomology, Hanoi, Vietnam; 2 Department of Translational Physiology, Infectiology and Public Health, Ghent University, Merelbeke, Belgium; 3 Department of Biomedical Sciences, Institute of Tropical Medicine, Antwerp, Belgium; 4 Department of Internal Medicine and Infectious Diseases, Ghent University Hospital, Ghent, Belgium; 5 Department of Epidemiology and Public Health, Sciensano, Brussels, Belgium; 6 Department of Public Health, Institute of Tropical Medicine, Antwerp, Belgium; University of Liverpool, UNITED KINGDOM

## Abstract

**Background:**

Fasciolosis is an emerging public health threat in a number of regions worldwide. To date, we lack an overview of both its occurrence and distribution in Southeast Asia across all actors involved in the life cycle, which impedes the development of disease control measures. Therefore, our objective was to collect recent information on the distribution and the prevalence of *Fasciola* spp. and the associated risk factors for infection in humans, animals, snails and plant carriers in Southeast Asia.

**Methodology:**

Bibliographic and grey literature databases as well as reference lists of important review articles were searched for relevant records published between January 1^st^, 2000, and June 30^th^, 2022. The systematic review was conducted according to the Preferred Reporting Items for Systematic Reviews and Meta-Analyses (PRISMA) guidelines for reporting systematic reviews. A total of 3,887 records were retrieved, of which 100 were included in the final analysis.

**Principal findings:**

The studies focused mainly on one host species (96.0%), with *Fasciola* spp. infection in animals being the most studied (72.0%), followed by humans (21.0%). Based on the used inclusion and exclusion criteria, reports were retrieved describing the presence of *Fasciola* spp. infection in seven out of 11 countries in Southeast Asia. Depending on the diagnostic tool applied, the prevalence of *Fasciola* spp. infection ranged between 0.3% and 66.7% in humans, between 0% and 97.8% in animals, and between 0% and 66.2% in snails. There were no studies reporting the presence of metacercariae on plant carriers.

**Conclusions/Significance:**

Our study reconfirms that *Fasciola* spp. infections are widespread and highly prevalent in Southeast Asia, but it remains difficult to accurately assess the true occurrence of *Fasciola* spp. in absence of well-designed surveys covering all hosts. As next steps we propose to assess the occurrence of the infection across all actors involved in the transmission, to identify associated risk factors and to estimate the burden of the disease to support national and international decision makers.

## Author summary

Fasciolosis is a foodborne disease that is caused by a parasitic worm. The disease affects both public and animal health worldwide. The transmission of *Fasciola* is complex and involves humans, animals and plants. The adult worms are observed in both humans and ruminants, while larval stages develop in freshwater snails. The infective larval stage attaches to aquatic plants. In recent decades, human fasciolosis has been considered an emerging disease in many regions of the world, including Southeast Asia. Although various studies surveyed *Fasciola* infection, an overview of existing knowledge on the distribution and prevalence of *Fasciola* across the different hosts is missing for this region. Yet, this is important to develop appropriate disease control measures. In this study, we systematically searched for literature on *Fasciola* spp. infection in Southeast Asia. Our study reconfirms that *Fasciola* is widespread in this region, but that it remains difficult to accurately assess the occurrence of parasite in the absence of well-designed surveys. A firm response to control fasciolosis requires both transdisciplinary and international partnerships. As next steps we propose to assess the occurrence of *Fasciola* spp. across all actors involved in the transmission, to identify associated risk factors and to estimate the disease burden to support national and international decision makers.

## Introduction

Fasciolosis is a parasitic disease caused by the zoonotic trematodes *Fasciola hepatica* and *F*. *gigantica* [1]. The transmission cycle of *Fasciola* spp. includes a final host (e.g., cattle, sheep, goats, buffaloes and humans), harboring the adult worms in the biliary ducts of the liver, an intermediate host (*Lymnaea* spp.) in which the larval stages develop and multiply, and a carrier (e.g. water plants) to which the metacercariae (i.e. the infective stage) are attached.

Fasciolosis poses a major threat to both animal and public health. In livestock, it is estimated that fasciolosis causes an annual global loss of 3.2 billion US dollars [2]. In humans, fasciolosis has recently emerged as a public health problem. Indeed, it is estimated that 2.4 million individuals are infected worldwide, with 180 million at risk in more than 70 nations [3], Bolivia, Peru, Egypt, Iran and Vietnam being the most affected countries [4]. To reduce the growing global disease burden, the World Health Organization (WHO) promotes large-scale administration of triclabendazole to individuals at risk in endemic countries (e.g., Bolivia, Ecuador and Peru, the Nile Delta in Egypt, and central Vietnam) [4]. However, due to the complexity of the transmission cycle of *Fasciola*, deworming programs aimed exclusively at humans may not be the most cost-effective strategy for long-term management of fasciolosis. Furthermore, there is an increasing number of reports of the ineffectiveness of triclabendazole against fasciolosis due to the development of resistance of the parasite to this drug, both in humans [5] and livestock, once again highlighting the urgent need for a transdisciplinary approach.

Indeed, a One Health strategy involving a wide range of specialties and sectors would be necessary [6]. Today, such a multisectoral strategy is often ineffective due to a lack of up-to-date information on the disease. For example, a case in point are the Southeast Asian countries (e.g. Thailand, Vietnam, Malaysia and Indonesia), where rice and water plants are important crops, and buffaloes and cattle are often kept alongside these fields [7]. In addition, the tradition of eating raw vegetables and using livestock manure to fertilize crops is still present in this region. In Southeast Asia, human fascioliasis is considered an emerging disease [8,9], and the economic impact of bovine fasciolosis is considerable, with annual losses estimated to range between 314 and 1884 million US dollars [7].

Although various studies surveyed *Fasciola* spp. infection in Southeast Asian countries, an overview of existing knowledge on the distribution and prevalence of *Fasciola* across the different hosts is missing, which impedes the development of a sustainable and integrated control strategy. The present study systematically searched literature on both (i) the geographical distribution and (ii) the prevalence of *Fasciola* infections in humans, mammals, snails, and water plants across Southeast Asia. In addition, we (iii) identified putative risk factors contributing to disease transmission across the different actors of the life cycle.

## Methods

This systematic review has been registered with the International Prospective Register of Systematic Reviews (PROSPERO), reference number: CRD42021261104. The detailed review procedures have been described elsewhere [10]. Briefly, records were retrieved from the bibliographic databases Cumulative Index to Nursing and Allied Health Literature (CINAHL), Excerpta Medica Database (EMBASE), PubMed, Scopus, and Web of Science (all databases), applying the following search phrase: (*Fasciola* OR fascioliasis OR fasciolosis OR *F*. *hepatica* OR *F*. *gigantica* OR liver fluke) AND (Southeast Asia OR Brunei OR Cambodia OR Indonesia OR Laos OR Malaysia OR Myanmar OR Philippines OR Singapore OR Thailand OR Timor-Leste OR Vietnam). Furthermore, grey literature was sought from the following sources: Asian Digital Library (http://www.theadl.com), the Index Medicus for South-East Asian Region (https://www.globalindexmedicus.net/biblioteca/imsear/), and the WHO Institutional Repository for Information Sharing (IRIS) (http://apps.who.int/iris/). Finally, reference lists of important review articles were screened for relevant records.

After merging the lists of the retrieved records, duplicates were deleted. In a first step, the relevance of the records was determined based on title and abstract screening. Subsequently, full-text articles were retrieved for selected records and examined for eligibility. Two members of the review team (VHQ and VD) evaluated the records independently for eligibility using a list of inclusion and exclusion criteria. Records were excluded based on the following criteria: (i) language not English, (ii) topic outside the research question (i.e. not covering the geographical distribution, the prevalence of and risk factors for *Fasciola* spp. in humans, animals or plant carriers), (iii) data from outside the study region (i.e. data not from Brunei, Cambodia, Indonesia, Laos, Malaysia, Myanmar, Philippines, Singapore, Thailand, Timor-Leste or Vietnam), (iv) data published beyond the study period (January 1^st^, 2000 and June 30^th^, 2022), (v) no full-text available or (vi) duplicate record. In case the independent eligibility evaluations of the two members of the review team were not in agreement, the records were jointly checked and thoroughly discussed until a consensus on the final evaluation for each article was reached.

**Fig 1:**
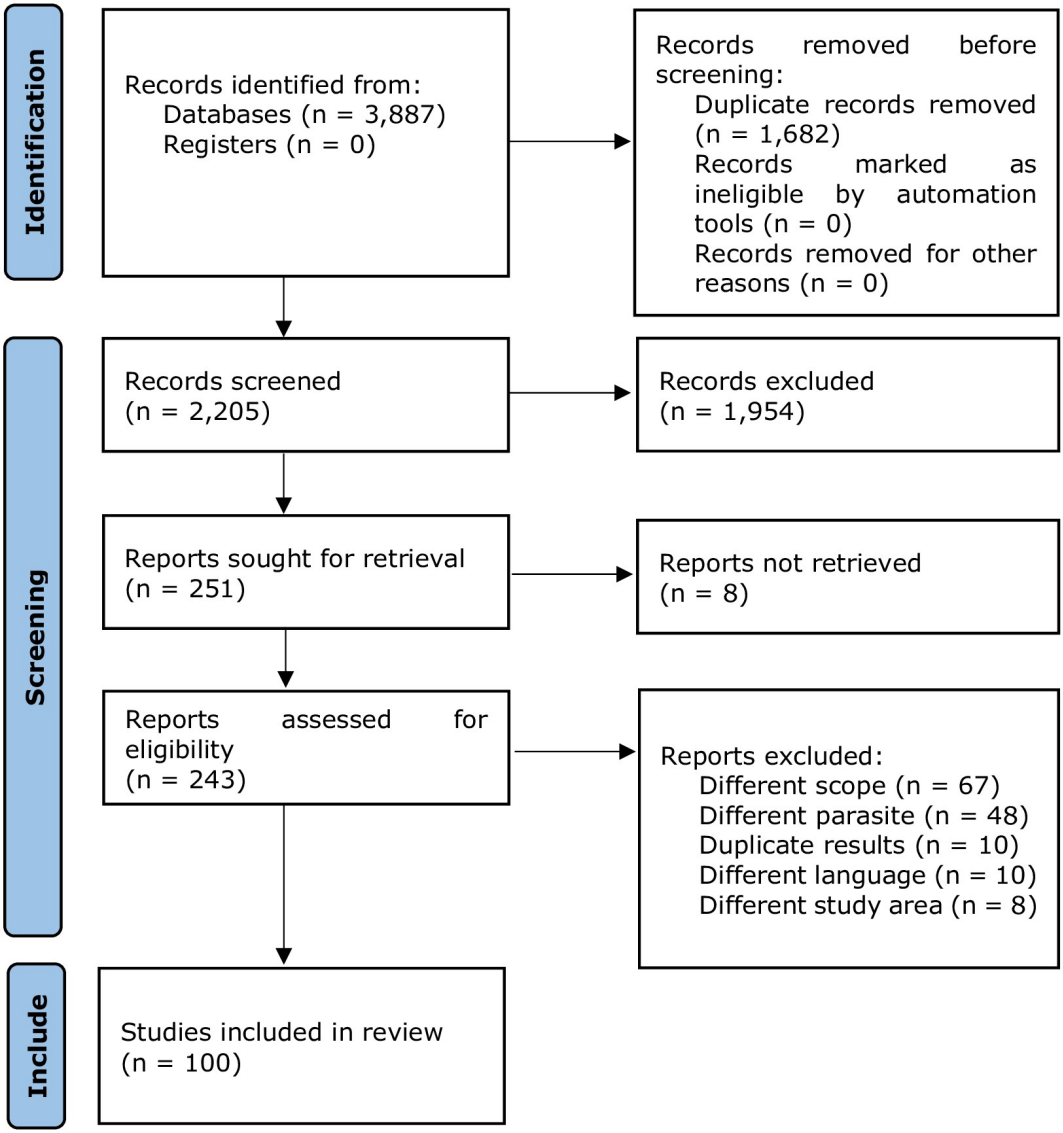
Flow diagram of the database searches according to PRISMA 2020 guidelines [11].

Data gathering forms were developed and tested to ensure relevant columns were included for the data we wished to extract. The data were retrieved by one of the review team members (VHQ), and a second team member then double-checked the data extraction (VD). In case the data extraction had to be edited by the second team member, a discussion was held until agreement was attained. From each retained record, author, reference, and publication year were extracted. For population studies, all information was extracted to allow for a a description of study design and sampled population, as well as for the estimation of the prevalence and associated 95% confidence interval (95%CI) (e.g., sample size and number of positives). Additionally, for cross-sectional studies and cohort studies investigating risk factors, numerators and denominators required to calculate odds ratios (OR) and associated 95%CI were extracted. For case reports and case series, general descriptors were extracted (e.g., age, gender, diagnostic tests applied). Generally, the review findings were reported in line with the Preferred Reporting Items for Systematic Reviews and Meta-Analyses (PRISMA) guidelines for reporting systematic reviews [11] (see Fig 1).

The statistical data analyses were run separately for each host type (i.e., humans, mammals, snails, plant carriers). Both for the population studies and case reports and series, a descriptive statistical analysis was conducted. For cross-sectional and cohort studies, the prevalence of *Fasciola* spp. was additionally calculated, as well as the associated Wilson score 95%CI. Odds ratios for the risk factors were calculated as well as associated Wald 95%CI. The significance was set at *p*<0.05. The statistical data analysis was performed in R 4.2.1 [12].

Finally, for each of the included population studies, the risk of bias was assessed by the (modified) Newcastle–Ottawa scale, which entails scoring for six different criteria [13,14]. A score for information on non-respondents was only given where relevant (i.e. in human studies, in the general population), while a score for the ascertainment of exposure and comparability was only given when risk factors were investigated in the study. The total score was calculated by summing all the criteria where the specific study had received a positive score, and dividing by the number of criteria for which a score was given. A study was given the high-quality label in case it reached a score of 100%.

## Results

A total of 3,887 records were retrieved from both the scientific databases and the grey literature sources (Fig 1). Following the removal of duplicates, the titles and abstracts of 2,205 records were reviewed. Subsequently, we retrieved the full text of 251 records and evaluated the eligibility. At this point, 151 records were excluded: 67 were omitted because the study topic was outside the scope of the systematic review; 48 records only reported data on other liver flukes, such as *Clonorchis sinensis* or *Opisthorchis viverrini*, or other parasites, such as soil-transmitted helminths; 10 records provided duplicate results, 10 records were not in English, eight records reported data from outside the study region, and for eight records a full text was not available. Finally, 100 records were included in the data analysis.

**Fig 2:**
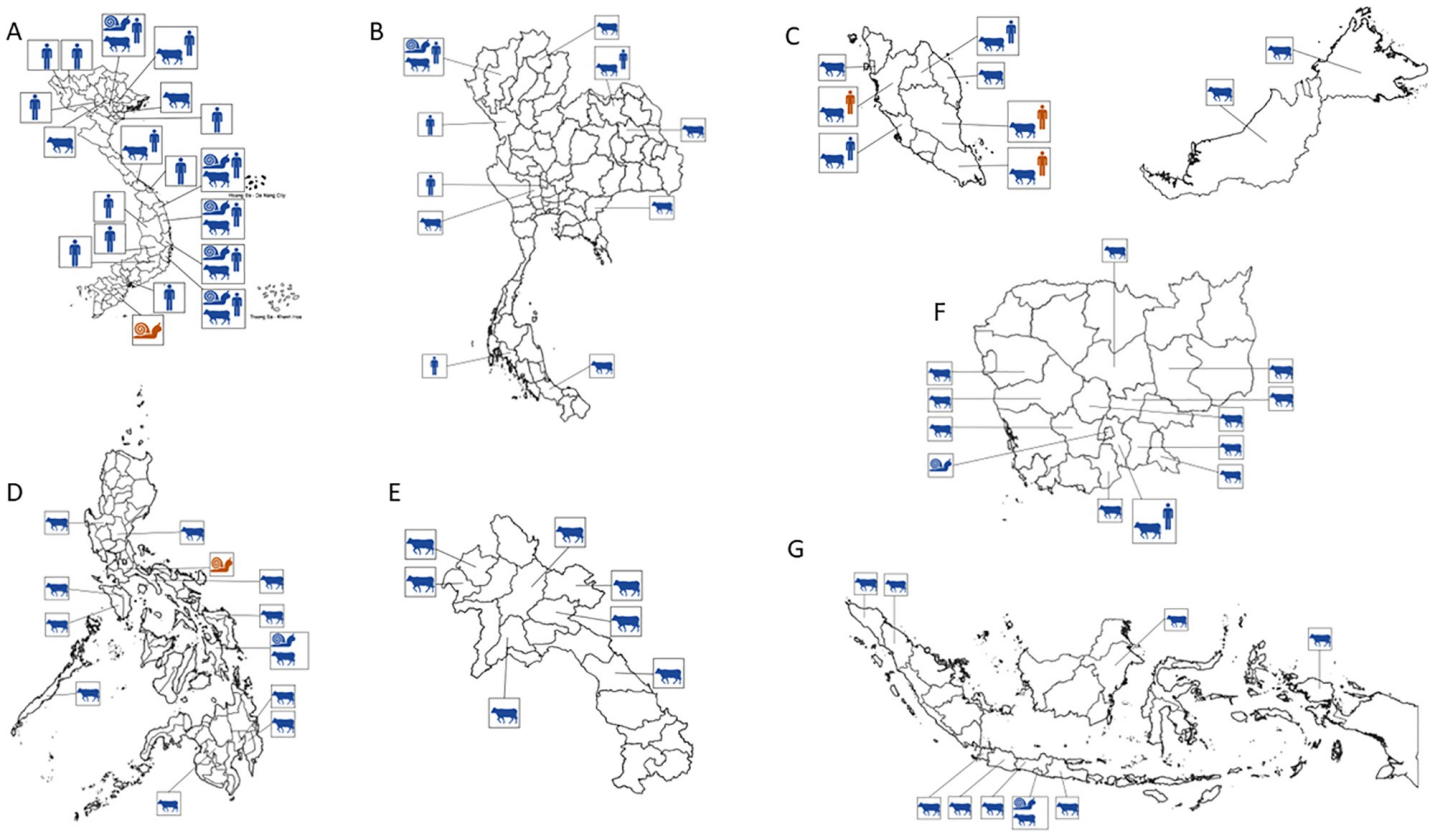
The geographical distribution of *Fasciola* spp. infection across the different Southeast Asian countries. Each of the 7 panels illustrates the distribution of *Fasciola* spp. infection in humans, animals and snails in Vietnam (Panel A), Thailand (Panel B), Malaysia (Panel C), the Philippines (Panel D), Laos (Panel E), Cambodia (Panel F) and Indonesia (Panel G). A blue silhouette indicates the presence of *Fasciola* spp., while a red silhouette indicates absence, for areas where no icons are shown, as well as for Brunei, Myanmar, Singapore, and Timor-Leste, no reports were found. Shapefiles were republished from the DIVA-GIS database (https://www.diva-gis.org/gdata) under a CC BY 4.0 license, with permission from Global Administrative Areas (GADM), original copyright 2018.

Based on the used inclusion and exclusion criteria, records were retrieved reporting the presence of *Fasciola* spp. in seven out of the 11 countries that are part of Southeast Asia: Thailand (n = 20), Vietnam (n = 19), Indonesia (n = 19), Malaysia (n = 13), the Philippines (n = 13), Cambodia (n = 10), and Laos (n = 6). For the remaining four countries (Brunei, Myanmar, Singapore, and Timor-Leste) no records were retrieved. The studies focused mainly on one host type (96.0%), with *Fasciola* spp. infection in animals being the most studied (72.0%), followed by humans (21.0%). In only a minority of the records, the presence of *Fasciola* spp. was assessed in snails (10.0%). One study reported *Fasciola* spp. on plant carriers, although not of the infectious life stage (1.0%). One study assessed *Fasciola* spp. infection in both cattle and snails [15], one in buffalo and snails [16], another screened both humans and snails [17], and one reported *Fasciola* spp. infection in humans and cattle [18]. The majority of the studies were cross-sectional studies (87.0%). The remaining 13 records were case reports. Fig 2 illustrates the geographical distribution of *Fasciola* spp. infection across the different Southeast Asian countries. In the following paragraphs we will summarize the findings in more details for each of host (humans, animals, and snails) and study type, separately.

### *Fasciola* spp. infection in humans

A total of 10 population surveys, representing 14 studies, investigated the presence of human *Fasciola* spp. infection in Vietnam (n = 7), Cambodia (n = 4), Malaysia (n = 2), and Thailand (n = 1) (Table 1). The studies involved different groups: school children (n = 5), hospital patients (n = 5), the general population (n = 2), and healthy adult subpopulations (n = 2). Antibody-Enzyme-Linked Immunosorbent Assay (Ab-ELISA) (n = 6) and copro-microscopy (n = 5) were the most deployed assays to diagnose *Fasciola* spp. infection. Other diagnostic methods used were copro-antigen-ELISA (copro-Ag-ELISA) (n = 1) and copro-Polymerase Chain Reaction (copro-PCR) (n = 1) [18]. One study did not mention the diagnostic method [19]. In the six studies that used the Ab-ELISA to detect antibodies against *Fasciola* spp., the prevalence ranged between 0.8 and 66.7%. In the five studies that used copro-microscopy, the prevalence ranged between 0.3 and 46.5%, while in the study that used a copro-PCR, the proportion of samples containing *Fasciola* DNA equaled 0.5% (Table 1).

**Table 1:** Human population surveys on *Fasciola* spp. infection in Southeast Asia.

Country	Population studied	Study Period	No. of people	No. positive	Prevalence (%)	95%CI	Test used	Species	Reference
***General population***									
Malaysia	Villagers	Nov 2007 to Jul 2009	716	2	0.3	0.03–1.01	Copro-microscopy	*Fasciolopsis*/*Fasciola*	[20]
Thailand	Population older than 15 years	May and Oct 2012	221	1	0.5	0.01–2.50	Copro-microscopy	*Fasciolopsis buski*/ *Fasciola*/*Echinostoma*	[21]
***Adult healthy subpopulation***									
Malaysia	Farm workers and dwellers	Dec 2017 to Dec 2018	90	60	66.7	56.4–75.5	Ab-ELISA	*Fasciola* spp.	[22]
Vietnam	Adult commune members	Mar and May 2013	1,612	125	7.8	6.55–9.16	Ab-ELISA	*F*. *gigantica*^a^	[17]
***Child healthy subpopulation***									
Cambodia	Primary school children	May and June 2011	228	106	46.5	40.1–53.0	Copro-microscopy	Large trematode eggs	[18]
Cambodia	Primary school children	May and June 2011	221	1	0.5	0.01–2.50	Copro-PCR	*Fasciola* spp.	[18]
Cambodia	Primary school children	May and June 2011	228	6	2.6	1.2–5.6	Copro-Ag-ELISA	*Fasciola* spp.	[18]
Cambodia	Primary school children	May and June 2011	240	2	0.8	0.1–3.0	Ab-ELISA	*Fasciola* spp.	[18]
Vietnam	Fifth grade children (14–15 years old)	Aug 2003 to Feb 2004	217	2	0.9	0.11–3.29	Copro-microscopy	Fasciolidae	[23]
***Patient subpopulation***									
Vietnam	Hepatic disease patients	2006–2007	143	37	25.9	19.4–33.6	Ab-ELISA	*Fasciola* spp.	[24]
Vietnam	Hepatic disease patients	2006–2007	143	3	2.1	0.4–6.0	Copro-microscopy	*Fasciola* spp.	[24]
Vietnam	Liver tumor patients	2006–2010	283	98	34.6	29.3–40.3	N/A	*Fasciola* spp.	[19]
Vietnam	Patients with serum samples submitted for *Fasciola* spp. antibodies	2012	10,084	590	5.8	5.41–6.33	Ab-ELISA	*Fasciola* spp.	[25]
Vietnam	Patients visiting the hospital for helminthiasis	2018	1,120	125	11.2	9.45–13.1	Ab-ELISA	*Fasciola* spp.	[26]

^a^As reported in the study, without reporting the method for species identification.

Two population surveys identified risk factors associated with human *Fasciola* spp. infection, these studies focused in patients and healthy adult populations in Vietnam and Malaysia (Table 2). In both surveys Ab-ELISA was deployed to diagnose *Fasciola* spp. infection. Being female, belonging to an older age group (at least 18 years in the study performed in Malaysia [22], above 60 years in the one in Vietnam [25]), and working with animals for a long period were identified as risk factors.

**Table 2:** Factors related with *Fasciola* spp. infection investigated in human population surveys in Southeast Asia.

Country	Variable	Comparison	Odds ratio (95%CI)	Reference
Malaysia	Age	≥18 years *vs*. <18 years	3.2 (1.1–9.8)	[22]^a^
Malaysia	Duration working with animals	≥ 5 years *vs*. <5 years	2.6 (1.1–6.4)	[22]
Vietnam	Gender	Male *vs*. female	0.8 (0.7–1.0)	[25]
Vietnam	Age	≤60 years *vs*. > 60 years	0.7 (0.5–0.9)	[25]

^a^Other factors investigated, but not significantly associated with infection: gender, education, monthly household income, drinking water treatment, consumption of raw vegetables, consumption of washed fruits and vegetables, consumption of fully cooked food.

In total, 872 fasciolosis cases were reported across 11 records in Thailand (n = 6), Vietnam (n = 4), and Malaysia (n = 1). There were 11 individual cases reported in six records (Table 3). On average these cases were 27.1 years old and seven of the 11 cases were female. Nine individuals reported abdominal discomfort (with five reporting abdominal pain and three reporting pain at the right upper quadrant), six patients reported fever, five cases lost weight, and four cases showed signs and symptoms such as, anorexia, chills, dark red serpentine track under the skin, and allergy. The remaining 861 fasciolosis cases were documented in five case reports series. In a record conducted in the south of Vietnam [27], 500 cases of fasciolosis were reported, with the majority of the patients (85%) being between 21 and 50 years old, and two-third being female. Another record described fasciolosis in 145 people diagnosed in a tertiary referral hospital in northern Vietnam [28]. Most of these cases (68.3%) were between 30 and 59 years old, and about half (51.0%) were male. The most frequently reported symptoms were upper quadrant (61.4%) and epigastric (35.2%) pain. In Thailand, fasciolosis cases were reported in three records, with the most common signs and symptoms being, abdominal pain (74.9%), especially in the right upper quadrant, abdominal distension (31.4%), weight loss (29.1%) in one record [29], while in another record [30] abdominal pain was present in all 12 patients, fever in three, and jaundice in one. Eleven patients had eosinophilia [30].

**Table 3:** Human fasciolosis case reports in Southeast Asia.

Country	Year of diagnosis	Sex	Age	Signs and symptoms	*S*pecies	Tests used	Reference
Malaysia	N/A	Male	56 years	Right hypochondrial pain for one month, chills and rigors	*Fasciola* spp.	Histopathology after mastectomy	[31]
Thailand	1985	Female	47 years	Multiple space-occupying lesions in the liver	*F*. *hepatica*^b^	Adult worm collection	[32]
	1999	Female	40 years	Abdominal mass for three months	*F*. *hepatica*^b^	Adult worm collection	[32]
	N/A	Female	67 years	Anorexia and weight loss for one month	*F*. *gigantica*^b^	Adult worm collection	[33]
	N/A	Female	36 years	Right-upper-quadrant abdominal pain with low-grade fever during the previous three months	*F*. *gigantica*^c^	Computed Tomography (CT)-scan, adult worm collection, and molecular assays	[34]
Vietnam	2000	Female	40 years	Burning pain at the right upper quadrant and a dark red serpentine track under the skin at the same site	*Fasciola* spp.	Adult worm collection	[35]
	N/A	Female	10 months	Fever (39 °C), upper abdominal pain, weight loss (0.5 kg), allergy	*F*. *gigantica*^c^	Ultrasound, stool (+), Ab-ELISA (+), and molecular assays	[36]
	N/A	Male	1 year	Fever (39–40 °C), upper abdominal pain, weight loss (0.7 kg)	*F*. *gigantica*^c^	Ultrasound, stool (+), Ab-ELISA (+), and molecular assays	[36]
	N/A	Female	3 years	Abdominal pain, chest pain, fever (38.5C) as first symptoms. Weigh loss (1.5 kg)	*F*. *gigantica*^a^	Ultrasound, stool (-), and Ab-ELISA (+)	[36]
	N/A	Male	3 years	Abdominal pain, cough, chest pain, fever (39 °C)	*F*. *gigantica*^a^	Ultrasound, stool (-), and Ab-ELISA (+)	[36]
	N/A	Male	4 years	Fever (39 °C) followed by abdominal pain appearing after two weeks and weight loss of around 2 kg/month	*F*. *gigantica*^b^	Ultrasound, stool (-), and Ab-ELISA (+)	[36]

^a^As reported in the study, without reporting the method for species identification;

^b^Based on morphometrics;

^c^Based on molecular analyses;

+: positive test result; -: negative test result

### *Fasciola* spp. infection in animals

A total of 70 population surveys were retrieved, describing 104 studies on *Fasciola* spp. infection in animals. These studies were conducted in seven countries, in Indonesia (n = 21), Malaysia (n = 18), the Philippines (n = 18), Thailand (n = 17), Cambodia (n = 11), Laos (n = 10), and Vietnam (n = 9). The most studied animal species were cattle (n = 67), buffaloes (n = 18), and both animal species surveyed in six studies. Other species examined were goats (n = 6), pigs (n = 2), deer (n = 1), monkeys (n = 1), leopard cats (n = 1), elephants (n = 1), and sheep and goats (n = 1). Copro-microscopy was the most commonly used diagnostic method, a total of 75 surveys used this diagnostic tool to demonstrate *Fasciola* spp. eggs in feces. Other diagnostic methods deployed were liver inspection at slaughter (n = 16), Ab-ELISA (n = 8), copro-PCR (n = 4) and copro-Ag-ELISA (n = 1). The prevalence across the different animal species and diagnostic methods are reported in Table 4 (copro-microscopy) and Table 5 (liver inspection, Ab-ELISA, copro-PCR and copro-Ag-ELISA).

**Table 4:** Animal population surveys on *Fasciola* spp. infection in Southeast Asia using copro-microscopic examination.

Country	Study Period	No. of animals	No. positive	Prevalence (%)	95%CI	Species	Reference
***Cattle***							
Cambodia	Dec 1998 to Nov 1999	575	142	24.7	21.3–28.3	*Fasciola* spp.	[37]
	1999 to 2000	N/A	N/A	N/A^a^	N/A	*F*. *gigantica*^b^	[38]
	Apr 2008	540	N/A	9.5	N/A	*F*. *gigantica*^b^	[39]
	July 2008 to May 2009	2,391	N/A	5.0–20.0	N/A	*Fasciola* spp.	[40]
	Sep 2008	540	N/A	21.5	N/A	*F*. *gigantica*^b^	[39]
	May to June 2011	205	182	88.8^c^	N/A	*Fasciola* spp.	[18]
	Dec 2014 to June 2015	172	28	16.3	11.5–22.5	*Fasciola* spp.	[41]
Indonesia	Nov 2011 to Jan 2012	394	49	12.5	9.54–16.1	*F*. *gigantica*^b^	[42]
	Feb to March 2016	369	129	35.0	30.3–40.0	*Fasciola* spp.	[43]
	Feb 2017	109	36	33.0	24.9–42.3	*F*. *gigantica*^b^	[44]
	Apr to Oct 2018	100	48	48.0	38.5–57.7	*Fasciola* spp.	[45]
	Jun to Sep 2018	314	113	36.0	30.9–41.4	*Fasciola* spp.	[46]
	2020	400	66	16.5	13.2–20.5	*Fasciola* spp.	[47]
	N/A	134	2	1.5	0.18–5.29	*Fasciola* spp.	[48]
	N/A	49	11	22.4	13.0–35.9	*Fasciola* spp.	[49]
	N/A	100	12	12.0	7.0–19.8	*Fasciola* spp.	[50]
	N/A	30	7	23.3	11.8–40.9	*Fasciola* spp.	[51]
	N/A	103	58	56.3	46.7–65.5	*F*. *gigantica*^b^	[52]
	N/A	35	13	37.1	23.2–53.7	*Fasciola* spp.	[53]
Laos	Sep 2010 to Feb 2011	172	20	11.6	7.6–17.3	*F*. *gigantica*^b^	[54]
	May and June 2015	21^d^	3	14.3	3.0–36.3	*Fasciola* spp.	[55]
	2016	241	95	39.4	33.5–45.7	*F*. *gigantica*^b^	[56]
Malaysia	Jan 2007 to Dec 2017	1,988	35	1.8	1.3–2.4	*Fasciola* spp.	[57]
	2008–2018	541	170	31.4	27.7–35.5	*Fasciola* spp.	[58]
	Mar 2015 to Jan 2016	219	111	50.7	44.1–57.2	*Fasciola* spp.	[59]
	Dec 2017 to June 2018	308	45	14.6	11.1–19.0	*Fasciola* spp.	[60]
	N/A	33	6	18.2	8.6–34.4	*Fasciola* spp.	[61]
Philippines	July to Sep 2011	45	44	97.8	88.4–99.6	*F*. *gigantica*^b^	[62]
	2017	15	5	33.3	15.2–58.3	*Fasciola* spp.	[63]
	N/A	250	N/A	27.1–49.0^e^	N/A	*F*. *gigantica*^b^	[64]
	N/A	80	13	16.3	9.7–25.8	*Fasciola* spp.	[65]
Thailand	Mar to Sep 2007	1,599	59	3.7	2.87–4.73	*Fasciola* spp.	[66]
	May 2013	322	34	10.6	7.7–14.4	*Fasciola* spp.	[67]
	2016 to 2019	1,501	35	2.3	1.7–3.22	*Fasciola* spp.	[68]
	Nov 2017 to July 2018	38	3	7.9	1.6–21.4	*Fasciola* spp.	[67]
	Jan and June 2019	311	26	8.4	5.8–12.0	*Fasciola* spp.	[69]
	Jan 2019	239	19	8.0	5.1–12.1	*Fasciola* spp.	[70]
	Aug to Nov 2020	333	67	20.1	16.2–24.8	*Fasciola* spp.	[67]
	Oct and Nov 2020	46	16	34.8	22.7–49.2	*Fasciola* spp.	[71]
	N/A	272	28	10.3	7.2–14.5	*Fasciola* spp.	[72]
Vietnam	Jan 1999 to Jan 2000	74	N/A	22	9.8–22.6	*Fasciola* spp.	[73]
	June 2002 to Mar 2003	157–204	N/A	0–31.0	N/A	*Fasciola* spp.	[74]
	June to Sep 2006	266	N/A	28.0–39.0	N/A	*Fasciola* spp.	[75]
	Apr 2008 to May 2009	1,075	487	45.3	42.3–48.3	*Fasciola* spp.	[15]
	June to Sept 2008	825	453	54.9	51.5–58.3	*Fasciola* spp.	[76]
	Nov 2010	153	89	58.2	50.2–65.7	*Fasciola* spp.	[77]
	Apr and Oct 2014	572	134	23.4	20.1–27.1	*Fasciola* spp.	[78]
***Buffalo***							
Indonesia	Mar 2017 to May 2018	580	93	16.0	13.3–19.2	*F*. *gigantica*^f^	[79]
Laos	May to June 2015	61^d^	1	1.6	0.4–8.8	*Fasciola* spp.	[55]
Malaysia	2008–2018	219	19	8.7	5.6–13.2	*Fasciola* spp.	[58]
Philippines	Apr 2006	15	3	20.0	4.3–48.1	*Fasciola* spp.	[80]
	July to Sep 2011	105	100	95.2	89.3–97.9	*F*. *gigantica*^g^	[62]
	Dec to June 2015 to 2016	335	213	63.6	58.3–68.6	*Fasciola* spp.	[16]
	Sep 2019	91	49	53.8	43.7–63.7	*Fasciola* spp.	[81]
	N/A	32	N/A	37.5–50.0	N/A	*F*. *gigantica*^b^	[64]
	N/A	48	12	25.0	14.9–38.8	*Fasciola* spp.	[82]
	N/A	80	15	18.8	11.7–28.7	*Fasciola* spp.	[65]
Thailand	May 2013	180	37	20.6	15.3–27.0	*Fasciola* spp.	[67]
	N/A	83	25	30.1	21.3–40.7	*Fasciola* spp.	[72]
	N/A	1,120	N/A	20.0–61.0	N/A	*Fasciola* spp.	[83]
***Cattle and buffalo***							
Cambodia	Dec 2001 to Jan 2002	1,406	160	11.4	9.8–13.1	*F*. *gigantica*^b^	[84]
	2004	801	103	12.9	10.7–15.4	*Fasciola* spp.	[85]
Laos	2010 to 2011	1,262	217	17.2	15.2–19.4	*F*. *gigantica*^b^	[86]
	Jan and Feb 2011	306^d^	81	26.5	21.8–31.7	*F*. *gigantica*^b^	[87]
***Goat***							
Indonesia	N/A	32	11	34.4	20.4–51.7	*Fasciola* spp.	[51]
Laos	May to June 2015	18^d^	1	5.6	0.1–27.3	*Fasciola* spp.	[55]
Malaysia	2008 to 2018	221	5	2.3	1.0–5.2	*Fasciola* spp.	[58]
Philippines	N/A	85	9	10.6	5.7–18.9	*Fasciola* spp.	[65]
***Sheep and goat***							
Malaysia	Mar to Dec 2015	267	0	0	0–1.4	*Fasciola* spp.	[88]
***Pig***							
Indonesia	N/A	4	1	25.0	0.6–80.6	*Fasciola* spp.	[51]
Philippines	Sep to Oct 2013	36^b^	1	2.8	0.07–14.5	*Fasciola* spp.	[89]
***Deer***							
Indonesia	N/A	75	20	26.7	18.0–37.6	*Fasciola* spp.	[90]
***Asian elephant***							
Malaysia	N/A	104	73	70.2	60.8–78.1	*Fasciola* spp.	[91]
***Proboscis monkey***							
Philippines	2011 to 2013	65	1	1.5	0.04–8.3	*Fasciola* spp.	[92]
***Leopard cat***							
Philippines	May 2013 to Feb 2014	3	1	33.3	0.8–90.6	Fasciolidae	[93]

^a^Reporting the village one-month incidence only, varying between 3.4 and 87.5%;

^**b**^As reported in the study, without reporting the method for species identification;

^c^Prevalence based on the combined used of copro-microscopic examination and copro-Ag-ELISA

^d^Prevalence represents the proportion of composite samples containing eggs;

^e^Prevalence based on the combined use of copro-microscopic examination and worm collection;

^f^Based on morphometrics;

^g^Based on molecular analyses;

**Table 5:** Animal population surveys on *Fasciola* spp. infection in Southeast Asia using other methods than copro-microscopic examination.

Country	Study Period	Diagnostic method	No. of animals	No. positive	Prevalence (%)	95% CI	Species	Reference
***Cattle***								
Cambodia	May to June 2011	Liver examination	191	35	18.3	13.5–24.4	*Fasciola* spp.	[18]
	July 2017	Liver examination	294	21	7.1	4.72–10.7	*F*. *gigantica*^a^	[94]
Indonesia	Feb to Mar 2019	Liver examination	100	39	39.0	30.0–48.8	*Fasciola* spp.	[95]
	N/A	Copro-Ag-ELISA	150	87	58.0	50.0–65.6	*F*. *gigantica*^c^	[96]
	N/A	Liver examination	150	92	61.3	53.3–68.8	*F*. *gigantica*^c^	[96]
	N/A	Liver examination	100	17	17.0	10.9–25.5	*Fasciola* spp.	[50]
	N/A	Liver examination	157	34	21.7	15.9–28.8	*F*. *gigantica*^b^	[97]
Laos	Sep 2010 to Feb 2012	Ab-ELISA	172	163	94.8	90.4–97.2	*F*. *gigantica*^c^	[54]
	July 2018 to Feb 2019	Copro-PCR	153	91	59.5	51.6–66.9	*Fasciola* spp. hybrids; *F*. *gigantica*^a^	[98]
Malaysia	2008–2018	Liver examination	1,128	19	1.7	1.1–2.6	*Fasciola* spp.	[58]
	Feb to Aug 2013	Liver examination	67	5	7.5	3.2–16.3	*F*. *gigantica*^b^	[99]
	Mar 2015 to Jan 2017	Ab-ELISA	85	70	82.4	72.9–89.0	*Fasciola* spp.	[59]
	Dec 2017 to June 2018	Ab-ELISA	308	115	37.3	32.1–42.9	*Fasciola* spp.	[60]
Philippines	July to Sep 2011	Copro-PCR	45	42	93.3	82.1–97.7	*F*. *gigantica*^a^	[62]
	2017	Liver examination	15	5	33.3	4.3–48.1	*Fasciola* spp.	[63]
Thailand	Oct 2010 to Sep 2012	Liver examination	51	27	52.9	39.5–65.9	*F*. *gigantica*^a^	[100]
	Oct and Nov 2021	Copro-PCR	46	31	67.4	53.0–79.1	*Fasciola* spp.	[71]
	N/A	Ab-ELISA	277	95	34.3	29.0–40.1	*Fasciola* spp.	[72]
Vietnam	Jun to Sep 2008	Ab-ELISA	400	289	72.3	67.7–76.4	*Fasciola* spp.	[76]
	Aor 2008 to May 2010	Ab-ELISA	235	205	87.2	82.4–90.9	*Fasciola* spp.	[15]
***Buffalo***								
Malaysia	2008–2018	Liver examination	245	2	0.8	0.1–2.9	*Fasciola* spp.	[58]
Malaysia	Feb to Aug 2013	Liver examination	13	1	7.7	1.4–33.3	*F*. *gigantica*^b^	[99]
Philippines	July to Sep 2011	Copro-PCR	105	101	96.2	90.6–98.5	*F*. *gigantica*^a^	[62]
Thailand	Oct 2010 to Sep 2012	Liver examination	55	37	67.3	54.1–78.2	*F*. *gigantica*^a^	[100]
	N/A	Ab-ELISA	95	75	78.9	69.7–85.9	*Fasciola* spp.	[72]
***Cattle and buffalo***								
Laos	Mar to June 2011	Liver examination	123	42	34.1	26.4–42.9	*F*. *gigantica*^c^	[87]
Malaysia	2018	Liver examination	7,786	25	0.3	0.2–0.5	*F*. *gigantica*^a^	[101]
***Goat***								
Malaysia	2008–2018	Liver examination	924	0	0	0–0.4	*Fasciola* spp.	[58]
	Mar to Dec 2015	Ab-ELISA	86	76	88.4	79.9–93.6	*Fasciola* spp.	[88]

^a^Based on molecular analyses;

^b^based on morphometrics;

^**c**^As reported in the study, without reporting the method for species identification;

Using copro-microscopy, *Fasciola* spp. infection rates varied from 0 to 97.8% in cattle and buffaloes, from 0 to 34.4% in goats, and from 2.8 to 25.0% in pigs. Based on liver inspection, the infection rates in cattle and buffalo ranged from 0.3 to 67.3%, while the prevalence ranged from 34.3 to 94.8% when the Ab-ELISA was used. When using copro-PCR, the prevalence ranged from 59.5 to 96.2%. Only one study applied copro-Ag-ELISA was to detect *Fasciola* spp. antigens in feces, with a reported prevalence of 58.0%.

Nine surveys reported data for factors associated with *Fasciola* spp. infection in animals, in Cambodia, Indonesia, the Philippines, Thailand and Vietnam (Table 6). The studies investigated *Fasciola* spp. infections in cattle or buffaloes, and all but two studies were based on copro-microscopic examination. Increasing age was a risk factor for cattle and buffalo in seven studies. Sex was found to be a risk factor in three studies, two of which determined that female animals had a higher risk of being infected. Furthermore, other risk factors for infection in cattle were flooding [41], the absence of deworming programs, not processing (i.e.) manure before further use, the presence of a high relative humidity, owners not participating in extension programs [47], and the rainy season [15], whereas in buffalo, the use of irrigation water for drinking was a risk factor [16]. Avoiding the use of cut and carry grass from a natural lake [41], treating animals [78] and access to wallowing ponds [16] were considered as protective factors in cattle or buffalo.

**Table 6:** Factors related with *Fasciola* spp. infection investigated in animal population surveys in Southeast Asia.

Country	Variable	Comparison	Odds ratio (95%CI)	Reference
***Cattle***				
Cambodia	Cut and carry grass from natural lake	No vs. yes	0.06 (0.01–0.5)^a^	[41]
	Flooding	Yes vs. no	20.7 (2.7–156.3)	[41]
	Age	≥ 3 years vs. < 3 years	3.5 (1.3–9.2)	[41]
	Sex	Female vs. male	8.1 (1.1–61.9)	[41]
Indonesia	Sex	Male vs. female	3.6 (1.6–8.3)	[52]
	Deworming program	No vs. yes	2.3 (1.2–4.2)	[47]
	Manure processed	No vs. yes	3.1 (1.6–5.9)	[47]
	Relative humidity	≥ 70% vs. < 70%	2.0 (1.1–3.5)	[47]
	Participation in extension program	Never vs. ever	2.6 (1.5–4.5)	[47]
Thailand	Age	> 7 years vs. ≤ 7 years	1.9 (1.1–3.4)	[72]
	Sex	Female vs. male	3.1 (1.5–6.2)	[72]
	Age	> 4 years vs. 2–4 years	3.5 (1.4–9.0)	[69]
	Age	> 4 years vs. < 2 years	3.2 (1.1–8.9)	[69]
Vietnam	Age	≥ 2 years vs. < 2 years	3.6 (2.7–4.9)	[76]
	Age	≥ 1 years vs. < 1 year	7.4 (4.6–12.1)	[76]
	Season	Rainy vs. dry	1.7 (1.3–2.2)	[15]
	Age	> 2 years vs. ≤ 2 years	1.9 (1.5–2.5)	[15]
	Treatment	Yes vs. no	0.09 (0.06–0.1)	[78]
***Buffalo***				
Indonesia Philippines	Age	> 32 months vs. 18–32 months	2.9 (1.2–6.8)	[79]
	Age	Per year increase	1.1 (1.1–1.2)^a^	[16]
	Wallowing pond	Yes vs. no	0.1 (0.08–0.2)^a^	[16]
	Irrigation water	Yes vs. no	10.4 (1.9–81.7)^a^	[16]

^a^Odds ratio (95%CI) from multivariable model

The number of case reports in animals was limited. One record described a fasciolosis case in a two-year-old male buffalo in Malaysia. In this case, *F*. *gigantica* adult worms were collected in the bile duct of the liver, and *Fasciola* eggs were found in the feces with a sedimentation test [102]. Another report described adult *Fasciola* in the liver of a deer in the Philippines [103].

### *Fasciola* spp. in the snail hosts and on plant carriers

A total of 11 records, describing 15 studies, assessed *Fasciola* spp. infections in snail hosts and on plant carriers. Fourteen studies investigated the prevalence of *Fasciola* spp. in snails in five countries (Table 7), including Vietnam (n = 7), Indonesia (n = 2), Thailand (n = 2), the Philippines (n = 2), and Cambodia (n = 1). The detection of *Fasciola* spp. infections in snails was mainly based on crushing of the snails followed by microscopic examination (n = 11). In two other studies, cercarial shedding was deployed, and in one, dissection. The prevalence of *Fasciola* spp. in snails ranged between 0 and 66.2% by crushing, and between 0.0 and 1.3% by shedding. *Fasciola* spp. were found in unspecified lymnaeid snails in four studies, and in *Austropeplea viridis/Lymnaea viridis* [15,104], *Lymnaea swinhoei* [15], and *Lymnaea rubiginosa* [105–107]. Two more studies reported the presence of *Gymnocephalous* cercariae, a cercarial type to which *Fasciola* spp. belong (among others), in *Bithynia siamensis* [108] and *Radix rubiginosa* [106]. There is one study reporting *Fasciola* eggs on Chinese cabbage, thus indicating the presence of fecal matter on this plant [109]. However, none of the retrieved studies reported the presence of *Fasciola* spp. metacercariae, the infectious stage, on plant carriers.

**Table 7:** Snail population surveys on *Fasciola* spp. infection in Southeast Asia.

Country	Study Period	Population studied	No of snails	No positive	Prevalence (%)	95%CI	Test used	Species	Reference
Cambodia	Mar 2007 to May 2008	*L*. *auricularia rubiginosa*^a^	79	N/A	1.3	N/A	Shedding	*Fasciola* spp.^b^	[106]
Indonesia	Apr 1993	*L*. *rubiginosa*	6,225	10	0.2	0.1–0.3	Crushing	*F*. *gigantica*^c^	[105]
	N/A	Lymnaeid snails	320	12	3.8	2.2–6.4	Dissection	*F*. *gigantica*^d^	[110]
Philippines	Dec 2015 to June 2016	Lymnaeid snails	748	495	66.2	62.7–69.5	Crushing	*Fasciola* spp.	[16]
	N/A	Lymnaeid snails	750	0	0	0–0.5	Shedding	*Fasciola* spp.	[111]
Thailand	Apr 2008 to Feb 2012	*Bithynia siamensis* ^e^	365	2	0.6	0.1–2.0	Crushing	*Fasciola* spp.^b^	[108]
	Apr 2008 to Feb 2012	*L*. *auricularia rubiginosa*	60	0	0	0.0–6.0	Crushing	*Fasciola* spp.^b^	[108]
Vietnam	Apr to May 2013	*Lymnaea* spp.	2,669	14	0.5	0.3–0.9	Crushing	*Fasciola* spp.	[17]
	N/A	*L*. *rubiginosa*	10,159	178	1.8	1.5–2.0	Crushing	*F*. *gigantica*^c^	[107]
	N/A	*L*. *viridis*	3,269	31	0.9	0.7–1.3	Crushing	*Fasciola* spp.	[15]
	N/A	*L*. *swinhoei*	1,128	7	0.6	0.3–1.3	Crushing	*Fasciola* spp.	[15]
	N/A	*Radix rubiginosa*	1,000	0	0	0–0.4	Crushing	*F*. *gigantica*^c^	[104]
	N/A	*Radix auricularia*	6,324	0	0	0–0.06	Crushing	*F*. *gigantica*^c^	[104]
	N/A	*Austropeplea viridis*	17,167	124	0.7	0.6–0.9	Crushing	*F*. *gigantica*^c^	[104]

^a^Seven other species were all negative;

^b^Based on the presence of gymnocephalous cercariae, a cercarial type to which *Fasciola* spp. belong (among others);

^c^As reported in the study, without reporting the method for species identification;

^d^Based on molecular analyses;

^e^Twelve other species were all negative

### Risk of bias

All of the 87 unique population surveys underwent risk of bias assessment. For 35 out of these (40.2%), the sample was truly or somewhat representative for the target population. For the seven studies where applicable, only one (14.3%) provided information on non-respondents. Data on risk factors were provided in 26 studies, 25 (96.1%) of which used a validated measurement tool for exposure, and eight (30.8%) controlled for other factors, by means of a multivariable model. A total of 79 out of 87 (90.8%) detailed the method used for outcome assessment. Finally, 56 out of 87 (64.4%) described the statistical methods. Only 17 studies (19.5%) were deemed to be of high quality.

## Discussion

Aligned with the sustainable development goal 3, which focuses on promoting good health and well-being, the WHO has pledged to eliminate the epidemics of diverse infectious diseases, including neglected tropical diseases of zoonotic origin, such as fasciolosis (WHO, 2020). To achieve these targets set by the WHO, it is crucial to have an in-depth understanding about the epidemiology of such diseases in regions where they are prevalent, in order to develop effective control strategies. While Southeast Asia is recognized as an endemic region for fasciolosis [112], a systematic, comprehensive and detailed assessment of the current epidemiological landscape across various hosts was lacking up to now. Thus, this study aimed to systematically review recent literature on the occurrence of and risk factors associated with *Fasciola* spp. in all hosts involved in its life cycle within Southeast Asia.

### Southeast Asia serves as a typical example of *Fasciola* spp. transmission

Our study analyzed population surveys from various sources, revealing prevalence estimates of up to 66.7% in humans and as high as 97.8% in cattle and buffaloes. Although investigated in fewer studies (13 out of 100 studies on the final animal host), the presence of *Fasciola* spp. was also detected in numerous other domestic (e.g., goats and pigs) and wildlife species (e.g., deer, monkeys and elephants) that consume plants in the region. Among the intermediate snail hosts, the prevalence ranged up to 66.2%. For the plant carriers, the absence of studies examining their contamination with *Fasciola* spp. metacercariae was notable, particularly considering their role as the source of infection for humans and animals. Although more studies are crucial in assessing the infection risk, the lack of quick and effective tools for assessing the contamination of plants with *Fasciola* spp. is hampering this investigation. Overall, the reported prevalence estimates for humans surpass those summarized in a similar review centered on Africa, which reported a prevalence range of up to 20.9% [113]. Similarly, prevalence estimates for animal final hosts in Southeast Asia are higher compared to those reported in East and Southern Africa, with estimates ranging up to 58% [114]. To the best of our knowledge, no systematic review has yet been published summarizing prevalence estimates for *Fasciola* spp. in the snail host and/or plant carrier, so comparisons are difficult for these life cycle actors.

Our systematic review also reconfirms the occurrence of human *Fasciola* spp. infection at the country level as previously reported by the WHO [112]. For Laos and the Philippines, however, we did not identify any cases in our review, which may be attributed to the fact that WHO may rely on the reporting of cases prior to 2000 (the earliest year covered in our review) or non-bibliographic government reports. For the animal final hosts, fasciolosis is not listed as a notifiable disease by the World Organization for Animal Health (WOAH) [115]. However, compared to the review article by Mehmood et al. 2017 [2], our study expands the description of *Fasciola* spp. infection occurrence in ruminants beyond just Vietnam and Cambodia to seven countries in Southeast Asia, which is line with the findings in the review of Calvani and Šlapeta 2021 [7]. The same applied for the snail hosts: while Xiao et al. 2018 [116] reported *Lymnaea viridis* and *Radix swinhoei* as potential transmitters of *Fasciola* spp. in Thailand and Vietnam, our systematic review expanded this knowledge by identifying records reporting the presence of *Fasciola* spp. in four snail species across four countries.

### Significant challenges remain to accurately assess the epidemiology of *Fasciola* spp. in Southeast Asia

Despite Southeast Asia being confirmed as an endemic region for *Fasciola* spp., our systematic review also revealed significant challenges in accurately assessing the epidemiology based on recent literature, primarily due to a dearth of well-designed studies. The risk of bias assessment conducted in our review indicated that less than a fifth of the population survey studies met the high-quality standards. The most commonly observed issues included the lack of information on non-respondents, not controlling for important factors in the analysis, and the lack of representativeness of the surveyed population compared to the national population. Consequently, the reported prevalence estimates may be subject to bias. Notably, certain studies also focused on specific subgroups such as, school children in Cambodia [18] and Vietnam [23], or hospital patients in Vietnam [19,24–26], potentially leading to an overestimation of the true underlying national prevalence. Moreover, there is a significant imbalance in the available literature, with a strong emphasis on specific host types. Among the different host species, the animal final hosts, particularly cattle, received the most attention, accounting for 46.2% (67 out of 145 studies) of the retrieved studies. Furthermore, a wide range of diagnostic tools were utilized in the studies, each with their own inherent limitations in terms of sensitivity and specificity. For instance, the microscopic examination of stool samples from the final human and animal hosts has a notoriously low sensitivity, due to the long prepatent period, low as well as intermittent egg shedding reported for *Fasciola* spp. [117]. The specificity can be hampered too in the presence of eggs of other trematodes such as, *Gastrodiscoides hominis*, *Paragonimus* spp., which are both prevalent in the study area. The Ab-ELISA, the most used technique for the human population surveys in Southeast Asia, on the other hand, is able to detect infections much earlier than copro-microscopy. However, the antigenic targets of the assays are often ill-defined, and the sensitivity and specificity not well characterized [118].

### Addressing the challenge of *Fasciola* spp. infections in Southeast Asia necessitates the establishment of more ambitious and transdisciplinary partnerships

Given the zoonotic nature of *Fasciola* spp., it is evident that comprehensive investigations incorporating One Health principles are crucial, especially in the light of more frequent reports on the parasites’ resistance to triclabendazole, up to now the drug of choice for mass drug administration campaigns [5]. However, our systematic review revealed a notable gap in literature, as none of the retrieved studies assessed the occurrence of *Fasciola* spp. across all actors involved in its life cycle (including humans, animals, snails, and plant carriers) within the same geographical area. Only four studies in our review combined the assessment of *Fasciola* spp. occurrence in two hosts (cattle and snails [15], buffalo and snail [16], humans and snails [17], and humans and cattle [18]. Moreover, none of the studies retrieved conducted a very thorough investigation of risk factors, such as the investigation of the effect of certain culinary, sanitation and animal management practices, although this is essential to understand the transmission of *Fasciola* spp. Season is another important, yet poorly studied factor, next to the above mentioned designed related factors, explaining the wide variability in prevalence, even within the same country. Indeed, infection with *Fasciola* spp. is thought to be seasonal, as appropriate temperature and humidity levels are needed for egg development and metacercarial survival, and lymnaeid snail population maintenance. Moreover, certain management practices, typical for Southeast Asia, such as allowing animals to graze on rice stubble after harvest might further contribute to the seasonality [7]. Finally, the presence of both *Fasciola* species, *F*. *hepatica* and *F*. *gigantica*, as well as their hybrids has been reported in Southeast Asia [7], but their exact distribution is ill-described, and the impact on pathology, transmission, prevention and control in the region are therefore poorly understood. Consequently, there remains a significant lack of scientific evidence necessary for the development of targeted intervention strategies. To effectively combat *Fasciola* spp., it is imperative to foster transdisciplinary collaborations that encompass all relevant hosts and incorporate a comprehensive exploration of risk factors, enabling the formulation of evidence-based interventions. In addition, the health impact of fasciolosis should be quantified by means of Disability Adjusted Life Years (DALY), an established WHO public health metric widely applied to express the burden of disease [119]. The socio-economic impact assessment should include direct as well as indirect costs related to human (e.g., costs for treatment; costs due to absence from work) and animal fasciolosis (e.g., costs for treatment and economic losses due to reduced meat and milk production and draught power, and condemnation of offal). Such an exercise would help in optimizing the allocation of resources towards the fight against this important disease.

Despite the strengths of our study in terms of conducting a systematic review comprising all the involved hosts in Southeast Asia, it is important to acknowledge certain limitations. Firstly, our language restriction may have resulted in the omission of relevant records published in languages other than English. This potential language bias could have impacted the comprehensiveness of our findings. Additionally, due to significant variations in prevalence estimates arising from differences in diagnostic tests, study designs, quality, and duration, we refrained from performing a meta-analysis to estimate national and regional prevalence of the disease. As is inherent to any systematic review, the estimated prevalence ranges are highly dependent on the quality of the included studies. Consequently, they may not accurately reflect the true prevalence ranges of *Fasciola* spp. infection in Southeast Asia, given the potential use of imperfect tests and limitations in the design of the studies included in our analysis.
